# Supplementary material for: A Humanized Yeast Phenomic Model of Deoxycytidine Kinase to Predict Genetic Buffering of Nucleoside Analog Cytotoxicity
Source: Genes (Basel). 2019 Sep 30;10(10):770. doi: 10.3390/genes10100770 (PMC6826991; doi:10.3390/genes10100770)
Supplement: Supplementary file 1 [file genes-10-00770-s001.zip › Additional_Files/Additional_File7_GOTermHeatmaps.zip!/Additional_File7_GOTermHeatmaps/intralumenal_vesicle_formation.pdf]

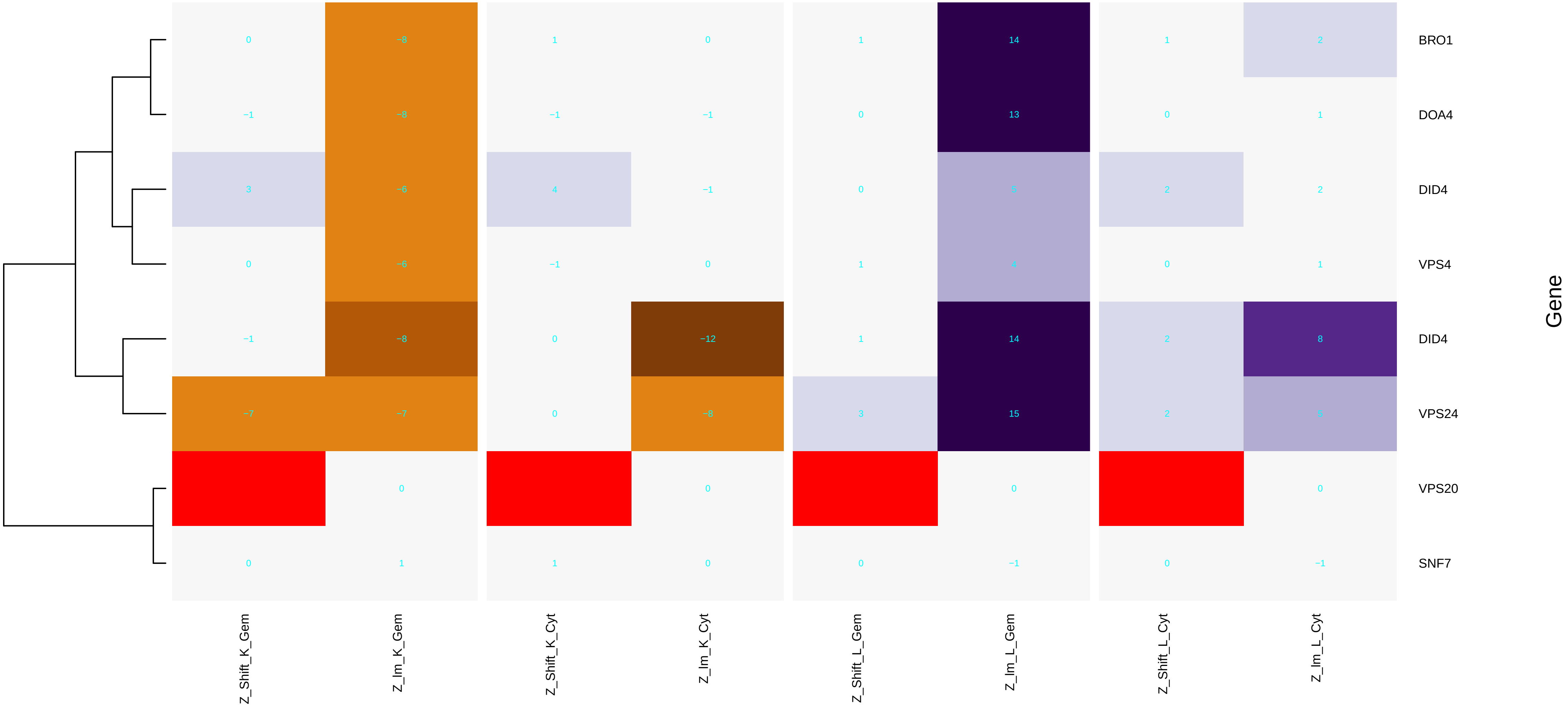

0 −8 1 0 1 14 1 2 BRO1
−1 −8 −1 −1 0 13 0 1 DOA4
3 −6 4 −1 0 5 2 2 DID4
0 −6 −1 0 1 4 0 1 VPS4
−1 −8 0 −12 1 14 2 8 DID4
−7 −7 0 −8 3 15 2 5 VPS24
0 0 0 0 VPS20
0 1 1 0 0 −1 0 −1 SNF7
Gene
Z_Shift_K_Gem
Z_Im_K_Gem
Z_Shift_K_Cyt
Z_Im_K_Cyt
Z_Shift_L_Gem
Z_Im_L_Gem
Z_Shift_L_Cyt
Z_Im_L_Cyt
